# Custom 3D fMRI Registration Template Construction Method Based on Time-Series Fusion

**DOI:** 10.3390/diagnostics12082013

**Published:** 2022-08-20

**Authors:** Zhongyang Wang, Junchang Xin, Huixian Shen, Qi Chen, Zhiqiong Wang, Xinlei Wang

**Affiliations:** 1 School of Computer Science & Engineering, Northeastern University, Shenyang 110819, China; 2 Key Laboratory of Big Data Management and Analytics, Northeastern University, Shenyang 110819, China; 3 College of Medicine and Biological Information Engineering, Northeastern University, Shenyang 110819, China

**Keywords:** fMRI template, fused slices, custom, sequences

## Abstract

As the brain standard template for medical image registration has only been constructed with an MRI template, there is no three-dimensional fMRI standard template for use, and when the subject’s brain structure is quite different from the standard brain structure, the registration to the standard space will lead to large errors. Registration to an individual space can avoid this problem. However, in the current fMRI registration algorithm based on individual space, the reference image is often selected by researchers or randomly selected fMRI images at a certain time point. This makes the quality of the reference image very dependent on the experience and ability of the researchers and has great contingency. Whether the reference image is appropriate and reasonable affects the rationality and accuracy of the registration results to a great extent. Therefore, a method for constructing a 3D custom fMRI template is proposed. First, the data are preprocessed; second, by taking a group of two-dimensional slices corresponding to the same layer of the brain in three-dimensional fMRI images at multiple time points as image sequences, each group of slice sequences are registered and fused; and finally, a group of fused slices corresponding to different layers of the brain are obtained. In the process of registration, in order to make full use of the correlation information between the sequence data, the feature points of each two slices of adjacent time points in the sequence are matched, and then according to the transformation relationship between the adjacent images, they are recursively forwarded and mapped to the same space. Then, the fused slices are stacked in order to form a three-dimensional customized fMRI template with individual pertinence. Finally, in the classic registration algorithm, the difference in the registration accuracy between using a custom fMRI template and different standard spaces is compared, which proves that using a custom template can improve the registration effect to a certain extent.

## 1. Introduction

In medical image registration, the two images involved in the registration are called the reference image and floating image, respectively [1,2,3]. Among them, the reference image will not change before and after registration. After registration, each meaningful anatomical point on the floating image will correspond to the corresponding anatomical point of the reference image one by one. Medical image registration is to find the corresponding optimal spatial transformation to make the anatomical points in the floating image consistent with the spatial position of the anatomical points in the reference image. The result of medical image registration should make the anatomical points of the two images, or at least all the points with diagnostic significance, match correspondingly [4,5,6].

The current brain image registration algorithm based on deep learning focuses on how to achieve spatial structure consistency between the image to be registered and the reference image of the same mode through transformation, which is more reasonable for the images to be registered from different subjects with high spatial resolution, such as MRI, which can be registered to the publicly available standard brain template for comparison, However, there is no standard template in the fMRI mode. In addition, for subjects whose brain structures are quite different from the standard template, such as the elderly and children, there will be a large error in registration to the standard space, and registration to the individual space can avoid this problem [7]. However, the individual template currently used is usually the fMRI image at a certain point in time selected by experts or researchers, which is highly accidental and depends heavily on the experience of researchers, which is not very reasonable.

As the brain standard template for medical image registration has been built only with an MRI template, there is no three-dimensional fMRI standard template to use, and when the brain structure of the subjects is quite different from the standard brain structure, registration to the standard space will lead to large errors, and registration to the individual space can avoid this problem.

For different subjects, there are often huge differences in the brain structures among the different individuals, and the spatial positions of corresponding points are obviously different. If not aligned, it is often impossible to carry out subsequent research and an analysis, such as determining the location of the focus by comparing the brain structure of patients with a disease with that of healthy controls [8,9,10]. Even for the same subject, due to the time interval of the image acquisition, different viewing angles, and different equipment, the corresponding points between the images will be different, such as the changes in the region of interest on the image before and after the time interval in the process of functional magnetic resonance imaging (fMRI) image scanning [11]. The emergence of medical image registration can well solve the above problems, effectively reduce the influence of position change during multiple imaging, and even correct the differences caused by the imaging mode itself [12].

At present, fMRI image registration algorithms can be divided into standard space-based registration and individual space-based registration [13]. Registration based on standard space is usually used for image registration among different subjects, and there is no published standard fMRI template at present [14], so most of them need to introduce the MRI standard template. However, it has great limitations for subjects with great differences in brain shape and structure from a standard brain, such as children and the elderly. Registration based on individual space effectively avoids this problem and is more suitable for fMRI data that usually help determine active brain areas by comparing the differences between the sequence data of the same subject. However, in the current registration methods based on individual space, most of the reference templates are randomly selected from data sequences, selected by researchers according to experience, or directly selected from the first effective time-point data, which leads to such methods being too dependent on researchers and having strong randomness [15]. Therefore, the registration of fMRI data needs a reasonable fMRI template based on individual space as a reference image for intra-individual registration.

Because fMRI can obtain four-dimensional data by scanning the brain many times in a time period, that is, a sequence composed of three-dimensional fMRI images at multiple time points, in which each three-dimensional fMRI image contains multiple two-dimensional slices corresponding to different layers of the brain, fMRI sequences often show the characteristics in large numbers and timing. Because fMRI speculates on the functional state of the brain by observing the active areas of the brain over a period of time, it is of great significance to change the position of features in images at different time points [16]. In order to keep as much information as possible in the registration of the 3D fMRI image sequences at different time points, it is necessary that the reference images used in the registration process contain more effective sequence information, so the template is constructed by using all effective fMRI data sequences at different time points.

As a group of fMRI image sequences generated by scanning the same position in a certain period of time by the functional nuclear magnetic resonance instrument, it often shows the characteristics in a large number, the orderly image itself, and the large correlation between two adjacent sequence images. The characteristics can indirectly reflect the movement trend of the object in the positions of different sequence images and have certain significance. The data at different time points have a certain correlation and do not exist independently. If we want to register a batch of fMRI images to a template, the more relevant feature information between the batch of images to be registered, the better. In order to make full use of the effective information between the fMRI sequences of the data to be registered, in the process of constructing the fMRI template, the transformation model is obtained by matching the two feature points of the two adjacent images based on the method of feature points. Then, by considering the relationship between the sequence data, the whole image is mapped to the reference image, so as to retain the effective information, such as the correlation between all the data, to the greatest extent, Finally, the matching results are fused to construct a custom fMRI template.

Therefore, a method for constructing a 3D custom fMRI template is proposed. First, the data are preprocessed. Second, by taking a group of two-dimensional slices corresponding to the same layer of the brain in three-dimensional fMRI images at multiple time points as image sequences, each group of slice sequences are registered and fused. Third, a group of fused slices corresponding to different layers of the brain are obtained. In the process of registration, in order to make full use of the correlation information between the sequence data, the feature points of each two slices of adjacent time points in the sequence are matched. Fourth, according to the transformation relationship between adjacent images, they are recursively forwarded and mapped to the same space. Finally, the fused slices are stacked in order to form a three-dimensional customized fMRI template with individual pertinence.

The contributions of this paper can be summarized as follows.

- A custom 3D fMRI template construction method based on time-series fusion is proposed to retain the correlation information in the time series to the greatest extent.
- The reference image is constructed to effectively improve the registration effect of the existing registration algorithm.
- Finally, the effectiveness of the proposed method is tested on a large number of real datasets.

The rest of paper is structured as follows. Section 2 introduces the custom template construction process. In Section 3, three groups of experiments are performed to verify the effectiveness of the proposed method. The purpose and results of the study are discussed in Section 4. Section 5 summarizes the methods and effects.

## 2. Method

**Figure 1:**
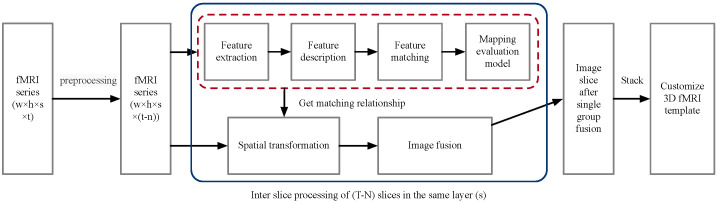
Custom fMRI template construction processes.

Referring to the method for constructing human brain template for fMRI, it can be seen that the construction process of human brain template usually includes three parts: image preprocessing, image registration, and image fusion. Therefore, the process of building a custom fMRI template in this chapter is shown in Figure 1.

Firstly, the fMRI dataset images are collected and preprocessed. The preprocessing steps include time layer correction and head movement correction. Time layer correction is used to correct the difference of acquisition time points between the middle layer and the layer in each three-dimensional brain process. Head movement correction is used to reduce noise interference. The preprocessed fMRI data can be regarded as multiple aligned three-dimensional brains. Secondly, for the registration and fusion of multiple groups of slices on the same layer in each three-dimensional fMRI image, the image registration method based on feature points is adopted. In the registration process, in order to make full use of the effective information between sequence data, the transformation model is obtained by matching two feature points of adjacent time-point slices, so as to retain the effective information between adjacent data as much as possible, and then by considering the relationship between sequence data, all effective slices are mapped to the same space by recursion. Next, the same layer slices of each group were fused to obtain a group of fused slices corresponding to different layers of the brain. Finally, the fused slices were stacked into three-dimensional custom fMRI templates in order.

### 2.1. fMRI Sequence–Feature Point Mapping Relationship Model

The registration method based on image feature points has the advantages of small amount of data, stability, and easy extraction. It can effectively and quickly process fMRI image sequences with large amount of data. Therefore, the image registration method based on feature points will be used to register fMRI image sequence data.

#### 2.1.1. Feature Point Extraction

Feature point extraction is the extraction and abstraction of the effective content in the image. It is the first link of image registration using feature points. Extracting stable, effective, and robust feature points is very important for image registration. Poor feature points or feature points extracted by noise interference will affect the determination of geometric–transformation relationship in subsequent registration. The feature points of fMRI slice images are extracted by SIFT method [17]. Sift method establishes multiple different scale spaces, obtains the key information such as the position and scale of feature points, and describes the point on the vector through these features, so as to achieve the purpose of extracting feature points. The process of extracting feature points using SIFT includes: constructing scale space, key point extraction, direction allocation, feature point description, and matching.

**Constructing scale space**: Convolving the original image with a two-dimensional Gaussian function at different scales can generate multiscale Gaussian space, the mathematical formula is expressed as follows.

(1) L(x,y,σ)=G(x,y,σ)∗I(x,y)

L(x,y,σ) represents multiscale Gaussian space. G(x,y,σ) represents Gaussian kernel function, I(x,y) represents image matrix. ∗ represents convolution operator, σ represents scale-space factor.

First, we downsample the image and Gaussian blur at different scales, establish multiple sets of multi-scale-space sequences to form the image Gaussian pyramid. Then, subtract adjacent images in each set of scale-space sequences, forming a difference of Gaussian pyramid (DOG) [18].

The mathematical expression is as follows.

(2) D(x,y,σ)=(G(x,y,kσ)−G(x,y,σ))∗I(x,y)

which leads to:

(3) D(x,y,σ)=L(x,y,kσ)−L(x,y,σ)

**Figure 2:**
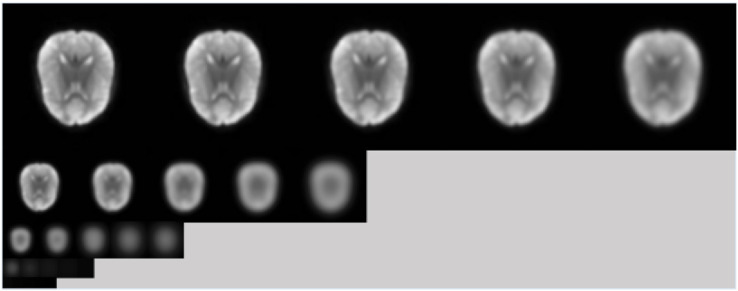
An example of Gauss pyramid.

**Figure 3:**
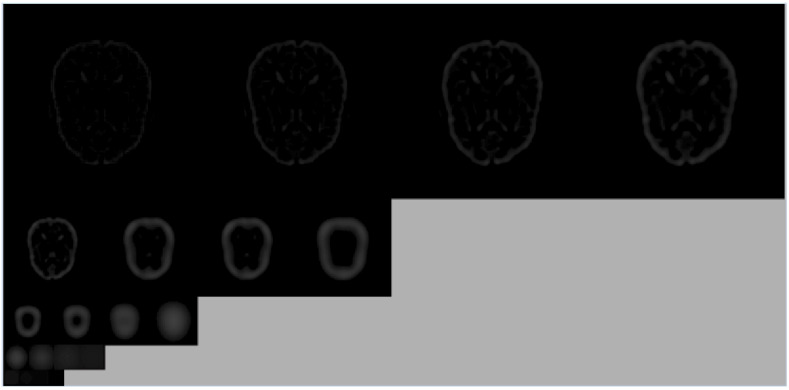
An instance of difference of Gaussian pyramid.

Figure 2 shows an example of a Gaussian pyramid. Figure 3 shows an example of the differential Gaussian pyramid. Although the image looks generally composed of black areas and can only see the subtle outline, it contains a lot of key information.

**Key point extraction**: Key point is the local extreme point of DOG. To form a local three-dimensional space, we compare the pixel at the core position with 8 same surrounding scale neighboring points and 9×2 different scale points with upper and lower neighboring points. Then, set the maximum or minimum point in each local stereo space as key point, fit the scale-space DOG function with a three-dimensional quadratic function in scale space, remove key points with poor stability.

(4) $$D\left(X\right)=D+\frac{\partial D^{T}}{\partial X}X+\frac{1}{2}X^{\tau }\frac{\partial^{2}D}{\partial X^{2}}X$$

$X={\left(x,y,\sigma \right)}^{T}$ and obtain the offset of extreme points as follows.

(5) $$\hat{X}=-\frac{\partial^{2}D}{\partial X^{2}}\frac{\partial D}{\partial X}$$

$\hat{X}={\left(x,y,\sigma \right)}^{T}$ represents the offset from the interpolation center. When the offset in any dimension is greater than 0.5, it means that the interpolation center has shifted, change the current key point position and interpolate at the new position to convergence. Then, obtain the precise location and scale information of feature points, find the principal curvature through Hessian matrix to eliminate unstable edge response points. The specific formula is as follows.

(6) $$\frac{\mathrm{Tr}(H)}{\mathrm{Det}(H)}=\frac{{\left(\alpha +\beta \right)}^{2}}{\alpha \beta }=\frac{{\left(r\beta +\beta \right)}^{2}}{r\beta }=\frac{{\left(r+1\right)}^{2}}{r}$$

The eigenvalues α and β of the *H* represent the gradients in the *x* and *y* directions. Tr(H) represents the sum of the diagonal elements of matrix *H*. Det(H) represents the determinant of matrix *H*. Assume α is the larger eigenvalue, and assume β is the smaller eigenvalue, let α=rβ, stable feature points will satisfy formula below.

(7) $$\frac{\mathrm{Tr}{\left(H\right)}^{2}}{\mathrm{Det}(H)}<\frac{{\left(r+1\right)}^{2}}{r}$$

**Figure 4:**
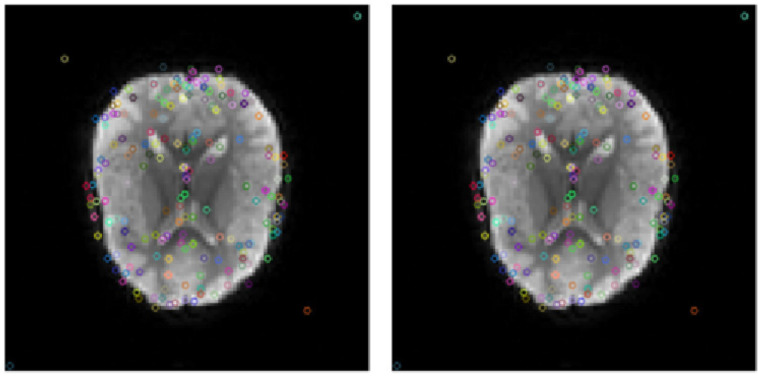
Examples of feature point extraction of $t_{0}$ image and $t_{1}$ image.

Keep the feature points that meet the above formula, otherwise eliminate them to complete the extraction of key points. The key point extraction results of fMRI slices at two different time points are shown in Figure 4. It can be observed that there are both the same key points, which can be matched as features, and different key points in the two images, which need further correction.

**Key points direction assignment**: In Gaussian pyramid image, we calculate the gradient modulus and direction of all pixels in a circular area with a radius of 3σ around each key point, use this as a reference to assign a reference direction for each key point. The magnitude and direction of the gradient are calculated, respectively. Using formula:

(8) $$m\left(x,y\right)=\sqrt{{\left(L\left(x+1,y\right)-L\left(x-1,y\right)\right)}^{2}+{\left(L\left(x,y+1\right)-L\left(x,y-1\right)\right)}^{2}}$$

(9) $$\theta \left(x,y\right)=\tanh^{-1}\frac{L(x,y+1)-L(x,y-1)}{L(x+1,y)-L(x-1,y)}$$

θ(x,y) represents the gradient direction angle of the feature point (x,y), m(x,y) represents the gradient modulus of the feature point (x,y), L(x,y) is the pixel value of the feature point (x,y) in the Gaussian pyramid. Set feature point as the center, rotate feature points within its neighborhood, keep the main direction at zero degrees. Then, use the gradient histogram to count the above information, divide the gradient direction into 36 columns, each column is divided by a span of 10 angles as abscissa, gradient magnitude as ordinate. Take the largest gradient amplitude as the main direction of feature points.

#### 2.1.2. Feature Point Description

To perform feature matching, a feature vector needs to be defined as the feature descriptor of each feature point as a unique “label” for each feature point. The feature vector can be regarded as an abstraction of the feature point and the information in the area is unique. Gradient location and orientation histogram (GLOH) [19] is used as a feature descriptor, as shown in Figure 4. We chose to use GLOH descriptors for the following reasons: Compared with PCA-SIFT and standard SIFT descriptors, it has better results on both edge features and smooth image processing. Secondly, compared with Speeded-Up Robust Features (SURF) [20], GLOH has a better processing result on blurred images. GLOH descriptor is robust and unique which reduces dependence on sample images. GLOH descriptors use a logarithmic polar hierarchy to replace the 4-quadrant traditional descriptor. Take a radius of 6, 11, 15 in space, and divide it into 8 intervals in angle (except for the middle area), and obtain 136 (17×8)-dimensional vector as the final feature vector.

#### 2.1.3. Feature Point Matching

Match every feature point of the reference image and every feature point of the image to be matched one-to-one. Calculate the Euclidean distance between the feature vectors corresponding to each pair of feature points, use this to determine the correspondence between them. The closer the Euclidean distance between two point feature vectors, the greater the chance of successful matching. The formula for calculating the Euclidean distance in n-dimensional space is as follows.

(10) $$d\left(x,y\right)=\sqrt{\sum_{i=1}^{n}{\left(x_{i}-y_{i}\right)}^{2}}$$

The specific method of feature point matching is as follows. First, select the feature point *B* in the image to be matched. Calculate the Euclidean distance between *b* and each feature point of the reference image and find the feature point with the smallest distance $B^{\prime }$. Then, find the nearest feature point *C* and the second nearest point *D* to point $B^{\prime }$ in the reference image. Calculate the ratio of the distance between points *B* and *C* to the distance between points *C* and *D* to obtain the correct matching point. The specific formula is as follows.

(11) $$\frac{d(B,C)}{d(B,D)}<\;\mathrm{Threshold}\;$$

The practical application in fMRI data is shown in Table 1. By comparing the number of matching successful feature points under different thresholds, this paper selects 0.9 as the given threshold.

**Figure 5:**
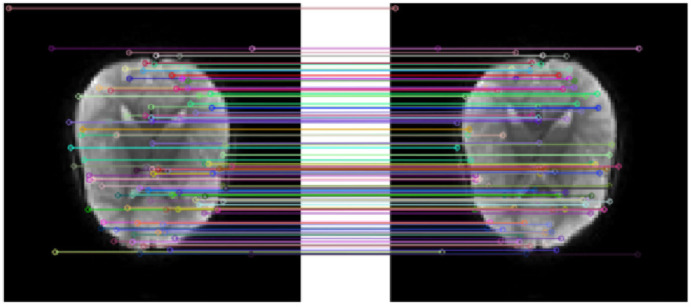
Example of feature point matching of $t_{0}$ image and $t_{1}$ image.

When the ratio is less than the given threshold (Threshold=0.9), the match succeeded; otherwise, it failed. An example of the feature point matching effect is shown in Figure 5.

#### 2.1.4. Mapping Model Evaluation and Image Sequence Matching

Through the above steps, the feature matching is realized, and the feature points matching each other in the two images are obtained. Then, this paper selects random sample consensus (RANSAC) algorithm to evaluate the mapping model and calculates the geometric–transformation relationship between the image to be registered and the reference image, according to the matching results. The transformation relationship between adjacent frames is obtained in two steps.

The specific implementation steps are as follows:

- Randomly extract four sample data from the feature point matching set M (the four samples cannot be collinear), calculate the transformation matrix, and record it as Model T;
- Calculate the projection error between all data in the feature point matching set M and Model T. If the error is less than the threshold TR, it will be added to the successfully matched feature point set G;
- If the number of elements of the current successfully matched feature point set G is greater than the optimal matched feature point set $G_{B}est$, then update $B_{B}est=B$, and the number of iterations K is updated at the same time;
- If the number of iterations exceeds the upper limit or the number of successfully matched feature points exceeds the critical value, exit the iteration; otherwise, the number of iterations is increased by 1, and steps 1–3 are repeated;
- Output the final transformation model T.

Set B is a set of matching point pairs that comply with epipolar constraints. The more elements in set B, the more accurate the estimation of the transformation model. RANSAC algorithm is to randomly select the matching items and ensure the transformation model with the largest number of elements in set B as the best result through iteration. The feature points that can adapt to the model results are called “local points”, and the feature points that do not adapt to the optimal model results are called “external points”. These external points may come from wrong measurement methods, wrong assumptions, wrong calculations, or extreme values of noise. In the process of iteration, the iteration round K is constantly updated rather than fixed when the number of iterations does not reach the limit value. The specific calculation method is as follows:

(12) $$k=\frac{\log(1-p)}{\log\left(1-w^{m}\right)}$$

where *p* represents the probability that the points randomly selected from the dataset are all local points in some iterative processes.

At this time, the model is likely to be useful, so *p* can also represent the probability that the results obtained by the algorithm are useful. As a confidence degree, it is usually taken as 0.995; *m* is the minimum number of samples required for the calculation model, which is 4 in this experiment; *w* represents the probability of selecting one local point from the dataset each time, as shown in the following formula:

(13) w=f/n

where *f* represents the number of local points and *n* represents the number of feature points of the dataset.

In the process of overall registration of fMRI image sequence, all images need to be mapped to the coordinate system of the same reference image. However, if each frame image is directly registered to the reference image, the correlation information between the front and rear adjacent frame images in the image sequence will be lost, which is quite fatal to the function-based sequence data of fMRI.

According to the comparison between the feature point matching similarity between the sequence image to be registered and the fixed reference image in Table 2 and Table 3 and the feature point matching similarity between adjacent frame images (only part is shown), it can be seen that the feature point matching similarity between adjacent frame images is generally higher than that of all images registered to the reference image at the first time point (after removing the data of the first n time points). This is also in line with the theory that there is a certain correlation between adjacent frame data, so the global registration of every two adjacent frame images is given priority. Firstly, starting from the transformation relationship between a pair of adjacent images, the relationship between each frame image and the previous image is recursively deduced. After the adjacent recursion is completed, the matching relationship between each image and the first time-point image is obtained according to the corresponding recursion relationship. It is assumed that $t_{i}$ is the matrix representation of the matching relationship between the relevant images between the i−th frame image and the i+1st frame image. The specific relationship representation is as follows:

(14) $$T=T_{1}\times T_{2}\cdots T_{i-1}\times T_{i}$$

### 2.2. Construction of Custom Template Based on Sequence Fusion

Next, each set of slices in the same layer after registration is regarded as a sequence, and the sequence is fused. According to the level of fusion process, image fusion algorithm can be divided into signal-level fusion, pixel-level fusion, feature-level fusion, and decision-level fusion. Among them, the signal-level image fusion fuses the unprocessed signal in the signal domain, and the fused signal is a random variable mixed with different correlation noise. This method can be regarded as a rough estimation of image fusion and cannot realize image fusion accurately. Feature-level image fusion extracts the feature information contained in the source image as the region of interest or target. However, because the feature information needs to be analyzed, processed, and integrated in the fusion process, many details are often lost. Decision-level image fusion is more targeted and less computational than the first two methods, but it is too dependent on the previous level, resulting in the blurred image. In contrast, pixel-level image fusion can retain as much information as possible in the source image. After fusion, the image has increased both in content and detail and is superior to other methods in accuracy and robustness. Therefore, pixel-level image fusion method is the most widely used in the field of medical images, and it is also a research hotspot in this field.

In the pixel-level fusion of the image, the direct average method is the simplest. It can directly sum and average the pixel values of the overlapping area, so as to avoid the problem that the overflow may lead to the failure of the normal representation of the image. Let f(x,y) be the fused image of $f_{1}\left(x,y\right)$ and $f_{2}\left(x,y\right)$, then the calculation expression of the direct average method is:

(15) $$f\left(x,y\right)\left\{\begin{array}{cc} f_{1}\left(x,y\right) & \left(x,y\right)\in f_{1}\\ \left[f_{1}\left(x,y\right)+f_{2}\left(x,y\right)\right]/2 & \left(x,y\right)\in \left(f_{1}\cap f_{2}\right)\\ f_{2}\left(x,y\right) & \left(x,y\right)\in f_{2} \end{array}\right.$$

Although the direct average method can perform simple and fast operations, the quality of the fused image is relatively poor because the direct average algorithm will weaken the contrast of the image, especially when the effective signal only exists in one image.

Therefore, the weighted average method is used to improve the direct average method, by weighting the two source images, rather than simply adding and summing to obtain the average. Let f(x,y) be the image generated after the fusion of $f_{1}\left(x,y\right)$ and $f_{2}\left(x,y\right)$, and the calculation formula of the weighted average method is:

(16) $$f\left(x,y\right)\left\{\begin{array}{cc} f_{1}\left(x,y\right) & \left(x,y\right)\in f_{1}\\ \omega_{1}f_{1}\left(x,y\right)+\omega_{2}f_{2}\left(x,y\right) & \left(x,y\right)\in \left(f_{1}\cap f_{2}\right)\\ f_{2}\left(x,y\right) & \left(x,y\right)\in f_{2} \end{array}\right.$$

In the above formula, $\omega_{1}$, $\omega_{2}$ are the weights corresponding to the pixel gray values of images $f_{1}$ and $f_{2}$ in the calculation process, and $\omega_{1}+\omega_{2}=1$, $0\leq \omega_{1}\leq 1$ and $0\leq \omega_{2}\leq 1$, the gradual-in and gradual-out method is generally adopted $\omega_{1}$ and $\omega_{2}$. Determine two weights:

(17) $$\omega_{1}=\frac{x_{2}-x_{i}}{x_{2}-x_{1}},\omega_{2}=1-\omega_{1}=\frac{x_{i}-x_{1}}{x_{2}-x_{1}}$$

where $x_{1}$ and $x_{2}$ are the left and right boundary coordinates of the part to be fused, respectively, Xi is the abscissa of the pixel to be fused, $x_{1}\leq x_{i}\leq x_{2}$. The weighted average method with added weight avoids the defects of the direct average method, and the operation is relatively simple, and the processing speed is very fast, so it is also widely used.

In addition, pixel-level fusion methods based on multi-scale transform, such as image fusion based on pyramid transform and image fusion based on wavelet transform, although they have their own uniqueness, the operation is more complex and takes longer time, which does not meet the short-term requirements of building a user-defined template for the subjects in this experiment, so they are not adopted. After the weighted average method is adopted to complete the sequence image fusion, a group of fused slices corresponding to the number of brain layers are obtained, and these slices are stacked in order to construct a three-dimensional custom fMRI reference template.

The custom template finally constructed in this paper retains as much information as possible in the sequence image, and the original size of fMRI sequence data is w×h×s×t, where *w* and *h*, respectively, represent the length and width of the scanned brain, *s* represents the number of layers of the brain scanned from top to bottom, and *t* represents the number of scans of the complete brain during the experiment. After preprocessing, the size is w×h×s×(t−n) four-dimensional fMRI sequence (the first *n* unstable time-point data are removed in the preprocessing process) converted into t−n three-dimensional fMRI sequences with the size of w×h×s NII format file, and then put each in 3D. The NII file is sliced into *q* two-dimensional files with the size of *w*, according to the *z*-axis. The DICOM format image of *h* saves the DICOM metadata of all slices regarded as the reference image at the first time point. Then, add a total of s×(t−n) DICOM data converted into PNG format, and t−n slices belonging to the same layer of the brain are matched and fused to form a set of two-dimensional slice images with the number of *s*. Then, dicom-concert software is used to convert the s PNG format slices into DICOM format, and the DICOM metadata of some important reference images, such as subject number and age, are embedded. Then, it will be stacked in three dimensions of NII format to become a user-defined reference template.

## 3. Experiments

### 3.1. Data

The data used in this article come from the Autism Brain Imaging Data Exchange (ABIDE) project [21]. This project aims to accelerate the understanding of the deep brain mechanism of autism spectrum disorder (ASD), which integrates the brain structure and functional imaging data from many laboratories around the world. The abide I and abide II datasets were used. The abide I data were collected from 17 centers, including 1112 subjects, including 539 ASD patients and 573 normal controls; the abide II dataset was collected from 19 centers, including 1114 subjects, including 521 ASD patients and 593 normal controls.

To construct a 3D custom fMRI template using fMRI time-series data, we first need to preprocess the data. The basic idea of fMRI data preprocessing is to eliminate the timing error of the interlayer scanning and the head movement error caused by the subject’s head movement in the scanning process, and then carry out the time-layer correction and head movement correction for the fMRI functional image. Because the custom template constructed for the subjects is eager to retain the individual specificity of the subjects, it does not need to be standardized. Therefore, the steps of the MRI structural image and the fMRI functional image registration and applying the corresponding MRI registration parameters to the fMRI functional images after the timing and head movement correction do not need to be introduced in the preprocessing process, and the data do not need to be smoothed. Therefore, this chapter only uses the time-horizon correction and head movement correction to process the data.

The SPM12 plug-in in the MATLAB 2020A environment is used for the data preprocessing in this experiment. The fMRI sequence registration algorithm based on the feature point extraction and the template construction algorithm based on image fusion can be applied to the template construction task of various single-subject medical image sequences. The relevant experimental environment is shown in Table 4.

### 3.2. Experiment Results

**Figure 6:**
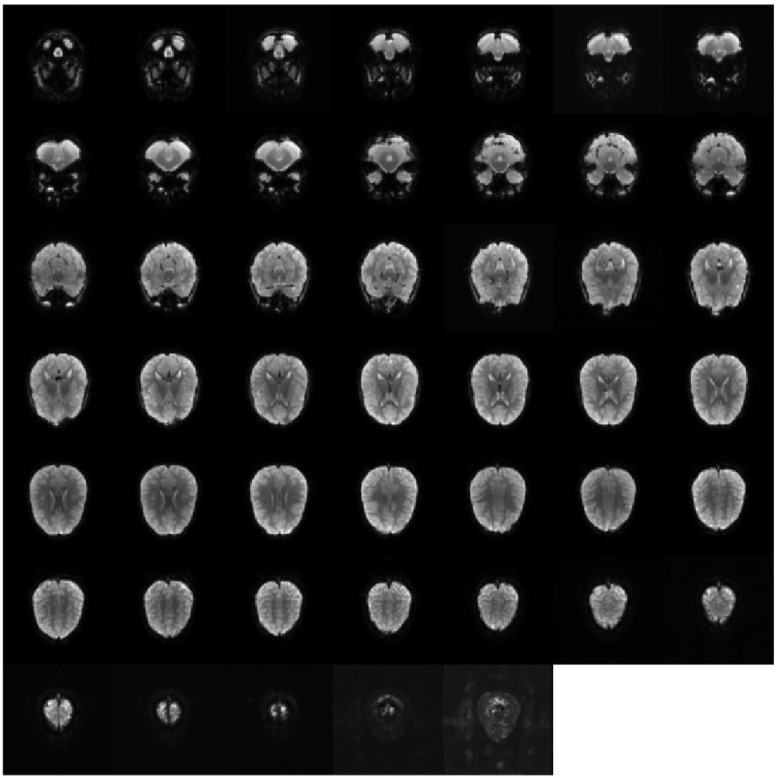
Display of custom template-instance slices of subjects of ABIDE50795.

Taking an 8-year-old girl with autism, numbered 50,795 in the Autism Brain Imaging Data Exchange (ABIDE) dataset, as an example, this paper analyzes the custom template in the form of slices. The fMRI data size of the subject is 96×96×47×156. After discarding the data images of the first four time points and eliminating the invalid data whose head movement exceeds the limit at two time points, the final effective data are 96×96×47×156, and the size of the final built custom template is 96×96×47, as shown in Figure 6.

**Figure 7:**
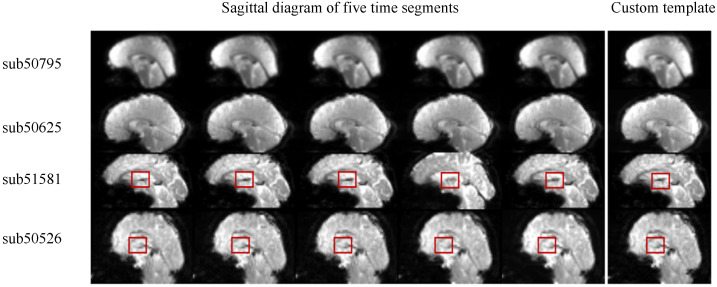
Display of customized templates for different subjects.

The custom template has individual pertinence, that is, it is constructed by using the subjects’ fMRI sequence. Therefore, the constructed custom template is related to the subjects’ age and brain structure, and the subjects are different from each other. Figure 7 shows the fMRI data of four example subjects with autism at five different time points and the custom built template in which sub50795 is an 8-year-old girl, sub50625 is a 7-year-old boy, sub51581 is a 64-year-old man, and sub50526 is a 50-year-old man. According to the sagittal diagram of the fMRI data of the four subjects, it can be found that there are very obvious differences in the brain structure, size, and development between the children and the adults. The user-defined fMRI template constructed for each subject retains the information contained in the images at different time points in the sequence data of the subjects to the greatest extent, especially for sub51581 and sub50526 of the two adults. Therefore, in the registration based on a single subject, it is more appropriate and reasonable to register the fMRI sequence data to the user-defined fMRI template than to randomly select the data at a certain time point as the reference image.

In order to verify the accuracy and rationality of the custom fMRI template, this paper uses several classical registration algorithms to evaluate the accuracy of the custom fMRI template. Under the two conditions of using the randomly selected time-point image as the reference image (in this experiment, the image of the first effective time point is directly used as the randomly selected time point) and using the user-defined fMRI template as the reference image, the registration accuracy of the fMRI sequence registration of the same subject is compared. In the experiment, the measurement value between the two images after the registration is calculated. And then, the average value is taken for display. Because both the image to be registered and the reference image are fMRI modes, the registration effect is evaluated by using the Mean Square Error (MSE), Advanced Normalized Correlation (NCC), Advanced Mattes Mutual Information (MI), and Normalized Mutual Information (NMI), commonly used to evaluate a single-mode registration. The matching criteria are to find the maximum correlation between the template and each subgraph, that is, the registered image. All the valid time-point data of 1114 subjects were registered with templates, and the average value of the evaluation indexes was displayed.

The smaller the mean square error (MSE), the better the registration effect. It can be observed from Table 5 that the self-defined fMRI template is used as the reference template for registration on the Affine, SyN of ANTs [22], and VoxelMorph registration algorithms based on deep learning [23]. The registration accuracy has been improved to varying degrees compared with using the randomly selected image at a certain $t_{0}$ fMRI volume (the first effective time point is directly used in this experiment) as the reference template for registration.

The registration based on a standard MRI structural image is the traditional method of fMRI registration. Because the spatial resolution of a fMRI image is relatively low, it is often difficult to define the similarity or difference between images intuitively, and when collecting fMRI data, the MRI structure image of the subject can usually be obtained at the same time, which has a high spatial resolution and richer structural details. Therefore, fMRI is registered with the help of an MRI image.

Most of the standard MRI structural images used in this process are selected from the standard human brain template database, mainly including the Talairach standard template [24], MNI305 standard template, ICBM152 template, and Colin27 template [25]. Among them, the Talairach standard template is corresponding to the Talairach standard space [24]; other brain templates correspond to the MNI standard space [26]. At present, the most commonly used standard MRI brain template is the MNI305 standard template, which is based on the brain structure of 305 adult subjects with an average age of 23.4 years. Here, the registration errors under the above standard templates are compared according to the Voxelmorph algorithm, as shown in Table 6. In the comparison experiment between the self-defined reference template construction algorithm for the fMRI sequence and the standard space method, which reduces noise interference, the correlation between the sequence images is preserved to the greatest extent. At the same time, the individual pertinence is retained, which effectively avoids the error caused by the large difference in shape and size when using the standard template to match the brains of patients, such as children and the elderly, and eliminates the contingency caused by the existing individual-based registration algorithm using randomly selected images at any time point as reference templates. The results show that the effect of a custom template is better than that of a standard template.

## 4. Discussion

At present, brain image registration algorithms focus on how to make the image to be registered consistent with the reference image in spatial structure through transformation. This is reasonable for images with high spatial resolution, such as MRI, from different subjects, which can be registered to publicly available standard brain templates for comparison, but there is no standard template with the fMRI mode at present. In addition, for the elderly, children, and other subjects whose brain structure is quite different from the standard template, there will be a big error in registration to the standard space, but registration to the individual space can avoid this problem. However, the individual template used at present is usually fMRI images selected by experts or researchers at a certain time point, which is accidental and depends on the experience of researchers, so it is not very reasonable.

Only an MRI template has been constructed for medical image registration, and there is no three-dimensional fMRI standard template available. When the brain structure of the subject is quite different from the standard brain structure, registration to the standard space will lead to large errors, but registration to the individual space can avoid this problem. However, in the current fMRI registration algorithm based on individual space, the reference image is often selected by researchers or randomly selected fMRI images at a certain time point, which makes the quality of the reference image depend on the experience and ability of researchers and has great contingency.

From the perspective of brain imaging, the imaging results of MRI and fMRI belong to two types of brain imaging, namely structural brain imaging and functional brain imaging. MRI is structural imaging, usually used in the study of the brain structure, such as for the diagnosis of brain tumors. MRI data are three-dimensional images. It can be regarded as infinite-time resolution, often with extremely high spatial resolution, and can provide abundant structural detailed information. In MRI brain imaging, we can distinguish the morphological structure of gray matter, white matter, and cerebrospinal fluid and also see the tiny details of various structures, such as anatomical boundaries, so as to judge whether there are lesions or injuries. fMRI data are a four-dimensional fMRI sequence with time dimension. As functional brain imaging, it is usually used to study cognitive and emotional processes. Imaging is obtained by scanning the brain several times over a period of time. fMRI specializes in studying blood flow in the brain and has very high time resolution, which is equivalent to the real-time log of the brain and is four-dimensional data containing time-dimension information.

Because of the inherent weakness of the MRI technology, the corresponding cost of high temporal resolution is the reduction in spatial resolution, so fMRI images cannot clearly observe the anatomical structure of the brain, so it is difficult to directly define the differences between the images.

According to the information contained in the image, the concept of fMRI-oriented medical image registration can be divided into two categories: function-based and structure-based. Among them, in the function-based fMRI image registration method, the registration based on the function signal needs to ensure the synchronization and consistency of different individual function signals, and many data are difficult to meet the requirements. The fMRI image registration method based on the global functional connection mode is based on the principle that the functional signals in the same fMRI data must be synchronized, consistent, and comparable. The whole brain–functional connection matrix is used to describe the functional information, but when some pixels in the matrix are disturbed by space, all points in the matrix will be affected, which leads to the lack of robustness of this method.

Because the function-based fMRI image registration methods require extremely high data and have strong application limitations, most of the existing fMRI image registration methods are structure-based. In this paper, fMRI registration is also based on structure, and the structure-based image registration is to align the floating image with the reference image in the anatomical structure. In order to ensure that the anatomical structures corresponding to the same voxel positions of two images are consistent, the structure-based registration first needs to map two images into the same space and use a common reference coordinate. There are two reference coordinate systems in the field of fMRI registration, which are standard space based on a large number of subjects and individual space based on a single subject.

fMRI registration based on standard space is to register the anatomical points of the brain images of subjects into the standard space constructed according to a large number of subjects. At present, the most common standard space is the MNI standard space. This method can be used for inter-individual registration or intra-individual registration. The images to be registered are registered into the standard space, and the standard human brain template is selected as the reference image, and then the differences between the images to be registered are determined by comparative analysis. There are two main methods for fMRI registration based on the standard space of a large number of subjects: registration based on standard MRI structural images and registration based on reference standard fMRI templates.

Registration based on standard MRI structural images is the traditional method of fMRI registration. Because the spatial resolution of fMRI images is relatively low, it is often difficult to define the similarity or difference between images intuitively. When collecting fMRI data, the MRI structural images of the subjects can usually be obtained at the same time, which has high spatial resolution and richer structural details. Therefore, fMRI registration is carried out by using MRI images. However, fMRI measures blood flow signals, while MRI measures tissue structure, which are of different orders of magnitude and cannot be directly compared. Moreover, due to the influence of noise and other factors, they may have different shapes. The advantage of this method is that it uses MRI images with high spatial resolution to estimate the deformation in standard space, but this method assumes that affine transformation can correct any difference between fMRI and MRI images of the same subject and does not take into account the influence of geometric distortion on fMRI data.

Most of the standard MRI structural images used in this process are selected from the standard human brain template database, mainly including the Talairach standard template, MNI305 standard template, ICBM152 template, and Colin 27 template. Among them, except that the standard space corresponding to the Talairach standard template is the Talairach standard space, other brain templates all correspond to the MNI standard space. At present, the most widely used standard MRI human brain template is the MNI305 standard template, which is constructed from the brains of 305 adult subjects with an average age of 23.4 years.

The registration of the reference standard fMRI template is to directly register the fMRI image to be registered with the selected three-dimensional standard fMRI template with low spatial resolution, which is the main problem solved by the single-mode registration based on depth learning at present. This method does not need MRI structural images, but because of the lack of adjustment of image details according to the MRI images, the registration results obtained are worse than the traditional two-step registration based on structural images, and there is no standard 3D fMRI template available publicly at present.

**Figure 8:**
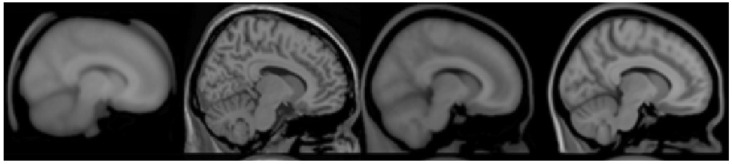
The sagittal plane of standard MRI templates: average305, colin27, ICBM152 T1, NLICBM152 T1 (from left to right).

**Figure 9:**
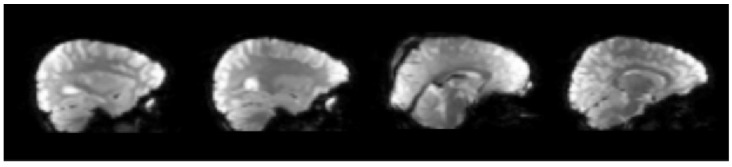
The sagittal plane of fMRI in children’s brains.

Brain image registration based on standard space selects the constructed standard space as the reference coordinate system. However, fMRI data are often used for the early diagnosis and treatment of mental diseases and many functional brain diseases, such as autism spectrum disorder and Alzheimer’s disease. Their onset period is often childhood or old age. As shown in Figure 8 and Figure 9, it can be found that the brains of such subjects are often quite different from the MRI standard general template made according to adult brains in size and brain structure, and the method of registering them into the standard space may cause large errors, which limits the application of this method to a certain extent.

In the analysis of a single-subject data sample, the above situation can be effectively avoided. Instead of normalizing the data into the standard space, the fMRI registration method based on the subject’s individual space is adopted, and the individual-specific fMRI template is used as the reference image. This method directly registers the image to be registered with the selected reference image, which is usually randomly selected by researchers from the image sequence, choosing a relatively standard fMRI image with a relatively standard shape and structure or choosing the fMRI image corresponding to the first effective time point as the reference image. The advantage of this method is that it retains the individual specificity of the reference image, which makes the registration based on a single subject more accurate. However, due to the randomness of the reference image selection or the dependence on the ability and experience of researchers, the method has great contingency.

A self-defined reference template construction algorithm for an fMRI sequence is proposed. The noise interference is reduced, the correlation between the sequence images is preserved to the greatest extent, and at the same time, the individual pertinence is retained, which effectively avoids the error caused by the large difference in shape and size when using the standard template to match the brains of patients, such as children and the elderly, and eliminates the contingency caused by the existing individual-based registration algorithm using randomly selected images at any time point as reference templates. By improving the rationality and accuracy of the reference template, the registration accuracy of the existing registration algorithm is further improved.

## 5. Conclusions

In this paper, a method for constructing a custom template for fMRI is proposed. Firstly, based on the acquisition of the ABIDE autism dataset, the preprocessing of the time-layer correction and head movement correction is carried out to prevent the data noise from affecting the quality of the custom fMRI reference image construction. Secondly, for the initial registration of the fMRI sequence images, the first time-point data are selected as the preliminary reference image, the feature points are extracted by the SIFT method, and the better GLOH descriptor is used to describe the feature points. After the evaluation of the feature points, the spatial–transformation relationship between the feature points is used to guide the geometric transformation of the fMRI sequence images to be registered. In this process, because there is a certain correlation between the fMRI adjacent frame images, in order to retain the correlation information of the adjacent frames to the greatest extent, give priority to obtain the corresponding matching relationship between the adjacent images, calculate the relationship matrix, then find the relationship between each time-point image and the reference image through recursion, and map all these images to the coordinate system of the reference image. Finally, the weighted average pixel-level fusion method is used to fuse the image sequence to obtain a user-defined fMRI reference template. In the classical registration algorithm, it is verified that in using the custom fMRI template instead of the random time-point image as the reference template, the registration accuracy has been improved to a certain extent.

## Figures and Tables

**Table 1:** Image matching quality evaluation.

Threshold of Distance Ratio	No. Feature Point Pairs Matched
1.0	109
0.9	113
0.8	105
0.6	100
0.4	93
0.2	70

**Table 2:** Similarity of feature points matching between sequence image to be registered and fixed reference image.

Reference	Image-to-Register	Feature Points of Reference Image	Feature Points of Image-to-Register	Number of Matching	Matching Similarity (%)
$t_{0}$	$t_{1}$	137	129	119	92.25
$t_{0}$	$t_{2}$	137	126	112	88.89
$t_{0}$	$t_{3}$	137	140	122	89.05
$t_{0}$	$t_{4}$	137	139	121	88.32
$t_{0}$	$t_{5}$	137	129	113	87.60
$t_{0}$	$t_{6}$	137	130	110	84.62
$t_{0}$	$t_{7}$	137	129	115	89.15
$t_{0}$	$t_{8}$	137	142	121	88.32

**Table 3:** Similarity of feature point matching between adjacent frame images.

Reference	Image-to-Register	Feature Points of Reference Image	Feature Points of Image-to-Register	Number of Matching	Matching Similarity (%)
$t_{0}$	$t_{1}$	137	129	119	92.25
$t_{0}$	$t_{2}$	129	126	114	90.48
$t_{0}$	$t_{3}$	126	140	113	89.68
$t_{0}$	$t_{4}$	140	139	125	89.93
$t_{0}$	$t_{5}$	139	129	122	94.57
$t_{0}$	$t_{6}$	129	130	116	89.92
$t_{0}$	$t_{7}$	130	129	118	91.47
$t_{0}$	$t_{8}$	129	142	119	92.25

**Table 4:** Related experimental condition.

Item	Version/Model
CPU	Intel 4210R × 2
GPU	Nvidia RTXA6000 × 2
Memory	256 G DDR4 ECC REG
Operating system	Ubuntu20.04
CUDA	11.2

**Table 5:** The comparison of registration results of registration algorithms.

Methods	Reference Image	MSE	NCC	MI	NMI
Affine	$t_{0}$ slice	0.91±0.27	0.57±0.21	0.62±0.27	0.61±0.13
	Custom template	0.88±0.20	0.59±0.18	0.63±0.13	0.64±0.15
SyN	$t_{0}$ slice	0.65±0.19	0.61±0.15	0.60±0.22	0.66±0.12
	Custom template	0.58±0.14	0.67±0.11	0.62±0.16	0.69±0.10
VoxelMorph	$t_{0}$ slice	0.78±0.22	0.52±0.23	0.63±0.21	0.63±0.14
	Custom template	0.73±0.17	0.57±0.12	0.65±0.27	0.68±0.12

**Table 6:** The comparison of registration results of standard human brain template.

Reference Image	MSE	NCC	MI	NMI
Talairach	0.83±0.21	0.53±0.19	0.66±0.21	0.63±0.33
MNI305	0.82±0.24	0.54±0.14	0.65±0.18	0.65±0.13
ICBM152	0.77±0.32	0.63±0.21	0.62±0.15	0.67±0.14
Colin27	0.72±0.20	0.56±0.19	0.64±0.23	0.66±0.19
Custom template	0.73±0.17	0.57±0.12	0.65±0.27	0.68±0.12

## Data Availability

The data are from the Autism Brain Imaging Data Exchange (ABIDE) project.
